# Treatment of Typhoid Fever

**Published:** 1854-07

**Authors:** Edwin R. Maxson

**Affiliations:** Adams Centre, Jefferson County, N. Y.


ART. II.—*Treatment of Typhoid Fever.* By Edwin R. Maxson, M. D., Adams Centre, Jefferson County, N. Y.

During a practice of eight years, in this locality, where Typhoid Fever prevails, to some extent, I have been led to the following conclusions, as to treatment, &c:

1st. That the disease is frequently propagated by contagion.

2dly. That whether contracted by contagion, or otherwise, the disease has a tendency to continue for two or three weeks, or longer, unless arrested by proper treatment.

3dly. That in most cases, the disease may be arrested, or broken up, if properly treated during the first stages.

4thly. That if neglected during the first stages, though it may not be broken up, the fever may be cut short materially, by proper medical treatment.

The following, is the plan of abortive treatment, which I have adopted, as most satisfactory.

I usually direct a warm foot bath, and friction along the spine, with an infusion of capsicum, in acid acetic.

℞ Capsicum, ʒii.

Acid acetic, Oss.

Infuse, and apply warm, with a flannel cloth. The foot bath appears to lessen the tendency to the brain, while the friction along the spine, equalizes the circulation, and thus relieves congestion, and consequently the distressing pain in the back, head, and limbs; while, both the foot bath, and the friction, have a tendency to, and will frequently produce, free perspiration. I usually direct, that both be continued, if necessary, morning and evening, for two or three days.

I usually give a mild cathartic, of hydg. cum creta, or hydg. chloride mite, in castor oil: or pill hydg. mass. gr. vi, followed in five or six hours, with oil. Warm drinks, of sage tea, and crust water, are allowed to be taken freely; and after the operation of the cathartic, toast is allowed, morning, noon, and night; and gradually other mild food.

And here, the fever is generally arrested; and convalescence begins, if seen within twenty four hours of the first attack.

If from being called later, or from any other cause, the fever still continues, I apply a blister to the epigastrium, and give

℞ Quinine sulph.
Pulv. Doveri aa. gr. iv.

Continued every four hours; and continue the crust water, till the fever is arrested, if that is accomplished within seven or eight days. If the fever continues beyond that time, I usually discontinue the Dovers and give camphor, with quinine.

℞ Pulv. camph. gr. i.
Quinine sulph. gr. iv.

Continued in mucilage of Gum Acc. every four or six hours.

I am satisfied, that in those cases in which the fever is not arrested by this treatment, in from three to eight days, that gastro-enteritis is generally the cause of its continuance: since, almost invariably, in my experience, any thing containing alcohol, as spts. æth. nit. though only a few drops be given, almost invariably and immediately increases the fever, and aggravates all the symptoms: while blisters over the stomach, (and bowels, too, if necessary,) is as speedily followed by less fever, and improvement of all the symptoms.

I have not spoken of other complications, for in my experience, if this treatment be early applied, they seldom occur; and if they do, should be treated on general principles; only that nothing should be given that could produce, or increase, gastro-enteritis, which so generally prevails in all protracted cases of continued fever.

